# Reported Barriers and Facilitators for Autistic Individuals, Persons with Other Intellectual and Developmental Disabilities, and Their Caregivers to Receive the COVID-19 Vaccine: A Pilot Study

**DOI:** 10.1007/s10803-024-06506-z

**Published:** 2024-08-16

**Authors:** Annie W. Resnikoff, Valerie Colantuono, Andrea Trubanova Wieckowski, Esther Chernak, Jennifer Plumb, Maurice Baynard, Elisabeth Sheridan, Diana L. Robins

**Affiliations:** 1 A.J. Drexel Autism Institute, 3020 Market Street, Philadelphia, PA 19104 USA; 2 Department of Psychology, Drexel University, Philadelphia, PA USA; 3 Drexel University Dornsife School of Public Health, 3215 Market St., Philadelphia, PA USA; 4 Academy of Natural Sciences of Drexel University, Philadelphia, PA USA

**Keywords:** Autism spectrum, Medical, Psychosocial or behavioral interventions, Sensory perception

## Abstract

Autistic individuals and persons with other intellectual or developmental disabilities (IDD) may experience challenges in social engagement, sensory processing, and behavior rigidity. This population is more likely to face barriers to successful preventative healthcare, including vaccines, compared to neurotypical peers. Autistic individuals and persons with other IDD may be at greater risk for COVID-19 infection due to sensory dysregulation that interferes with mitigation such as wearing masks, and challenges in social communication that impose difficulties in understanding and adhering to prevention measures. Adaptations are needed to make vaccine opportunities more accessible for neurodivergent individuals. A series of seven Sensory-Friendly COVID-19 Vaccine Clinics (SFVCs) were conducted between December 2021 and August 2022 in collaboration with the A.J. Drexel Autism Institute and the Academy of Natural Sciences of Drexel University. SFVCs examined perceived barriers and facilitators to vaccine experiences, based on feedback from autistic individual/persons with IDD and their caregivers. Surveys were administered to autistic individuals/persons with IDD or their caregivers (n = 35) from the larger sample who attended the clinic; 18 participants also complete a supplemental interview. Scaled survey questions were analyzed to determine the acceptability of the SFVCs. Open-ended survey questions and interview responses were coded thematically to identify barriers, facilitators, and areas of improvement. All individuals who came to a SFVC with intent to be vaccinated were successfully administered a COVID-19 vaccine. More than 90% of participants reported that experiences at the SFVCs were positive, promoted retention, and they would recommend clinics to others. Staff clinical expertise, sensory-friendly elements, and hosting clinics at a neutral location (free from past medical history) served as facilitators to successful vaccine administration, whereas factors such as ill-equipped pharmacy staff, behavioral challenges, and logistical issues may serve as barriers. Incorporating reported barriers, facilitators, and accommodations of SFVC experiences may lead to more successful preventative healthcare processes for neurodivergent individuals.

## Introduction

Note on terminology. Although perspectives on person-first vs. identify-first language vary among stakeholders, identity-first language (e.g., autistic person) is intentionally used throughout this paper, as it aligns with recent autistic self-advocate preferences (Taboas et al., 2022).

Autism is a neurodevelopmental condition that affects social skills, communication, learning, and behavior. Approximately 1 in 36 children are diagnosed with autism (Maenner et al., 2023), and approximately 1 in 6 children have at least one intellectual or developmental disability (IDD) diagnosis, including autism (Zablotsky et al., 2019). As the population of autistic individuals and those with other IDD grows, access to equitable preventive healthcare, such as vaccinations, should expand to meet their needs.

Autistic individuals and their families experienced unique challenges in response to the COVID-19 pandemic, including in their educational/vocational services, home and recreational activities, access to behavioral health services, and changes in health service delivery (Baweja et al., 2022). Up to 95% of autistic children have impairments in some sensory modality and these manifestations tend to perpetuate in adulthood (Tomchek & Dunn, 2007). Both hypo- and hyper-sensory sensitivities contribute to increased anxiety, specifically regarding the sensory aspects of unpredictable situations, such as vaccine administration (Hwang et al., 2019). Common sensory sensitivities experienced in medical settings include noise from other patients, bright or fluorescent lighting, and unexpected touch or smells (Doherty et al., 2022). Challenges in social communication may also lead to difficulties in understanding and adhering to collective and individual prevention measures (Courtenay & Perera, 2020).

Of critical importance, autistic individuals and others with IDD are at increased risk for becoming infected with COVID-19, are more likely to experience post-COVID conditions (Liu et al., 2023), and may also face substantial barriers to vaccination (Schott et al., 2021). Although difficulties are not isolated to the COVID-19 vaccine, barriers may be exacerbated due to the medical necessity and timeliness of the COVID-19 vaccine schedule. The COVID-19 pandemic brought a unique urgency of vaccine administration, which hindered autistic individuals and other individuals with IDD ability to prepare in ways that were historically successful. Time did not allow for processes such as repeated exposures, identification of functional reinforcers, or pre-visit care planning to assess individuals' specific needs, all of which have been identified as best practices for autistic children in the hospital setting (Call et al., 2022). Additional potential vaccine barriers range from inability to schedule an online appointment or secure transportation (Ryerson et al., 2021), uneasiness in the waiting area and/or vaccination space prior to and during vaccine administration (e.g., noise from other patients, crowded area, uncomfortable furniture), feeling misunderstood by healthcare providers, as well as past negative experiences with healthcare providers (Doherty et al., 2022). It may be difficult to find medical providers confident in working with autistic individuals or others with IDD, as many healthcare professionals report feeling uncomfortable treating this population (Ghaderi & Watson, 2019). Additionally, there may be caregiver hesitation to vaccinate their children, due to concerns about vaccination contributing to autism (Thorsteinsson et al., 2020) despite the absence of scientific data supporting these concerns (Madsen et al., 2003; Taylor et al., 1999). Caregivers of autistic children report a higher rate of vaccine skepticism as a barrier compared to caregivers of neurotypical youth (Golnik et al., 2009).

Despite having more frequent healthcare visits (e.g., primary care, specialty care, acute care) than neurotypical individuals, autistic youth are less likely to receive preventative healthcare, including vaccinations (Cummings et al., 2015). Previous literature has primarily focused on barriers to care. Accommodations and adaptations that make vaccination opportunities more accessible to persons of all ability types, including autistic individuals or persons with IDD, may serve as facilitators to successful preventative healthcare services, including vaccinations. There is no known set of best practices or standards of preventive care for autistic individuals, however several potential facilitators to successful preventative healthcare visits, including vaccine administration, have been identified. Facilitators to care include flexibility of practice to accommodate patient needs whenever possible (e.g., performing routine exams in a chair instead of an exam table), including the autistic individual in healthcare decisions, seeking clinicians that are equipped with autism-specific training and briefed on the individual’s profile (e.g., their triggers, sensitivities, communication style etc.), and access to longer appointment times (O’Hagen et al., 2023). Additional adaptations may include offering vaccine clinics at a neutral space where there is no history of negative medical experiences, low-stimuli spaces, calm waiting areas, clinical support by trained professionals, increased transition times, privacy for individuals and their families, and access to activities or stress-reducing tangible items pre- and post-vaccine administration. While these adaptations are intended to produce a more comfortable environment that facilitates vaccination of autistic individuals, there are few data regarding the experiences of these individuals or their caregivers in these settings, and the degree to which these “sensory friendly” vaccination sites facilitate vaccination or improve the clinical experience of vaccination.

The objective of this pilot study was to understand the experience of vaccine recipients using a sensory-friendly vaccine clinic (SFVC) approach and identify practices to improve the vaccination experience of autistic individuals and those with other IDD, via survey and interview conducted with vaccine recipients and/or their caregivers.

## Methods

### Study Design and Setting

During the height of the pandemic, COVID-19 vaccination programs relied heavily on high through-put mass vaccination clinics, and commercial pharmacies to provide vaccines to the public. Recognizing that these venues might be challenging for autistic individuals, the A.J. Drexel Autism Institute (AJDAI) collaborated with the Academy of Natural Sciences of INSTITUTION (ANS), Sunray Pharmacy, and staff from the INSTITUTION College of Medicine to host and operate SFVCs geared for autistic individuals or persons with other IDD. The study received Institutional Review Board approval from INSTITUTION.

To identify facilitators and barriers to vaccination presented by this model, we surveyed a subset of COVID-19 vaccine recipients and/or their caregivers from these SFVCs. A total of 7 SFVCs were operated at the ANS, a natural history museum, from December 2021 to August 2022. The SFVCs occurred approximately 3–6 weeks apart, on Saturday or Sunday mornings, and were typically open from 9 am to 12 pm. There was variability in clinic attendance, ranging from 2 to 50 attendees per session. Clinic staff with autism expertise, comprising psychologists, physicians, pharmacists, behavior analysts, trainees, and clinic volunteers, provided supports throughout all clinic processes. Clinic space was arranged within a large and open room in the museum building, and consisted of three sections: (1) an entry/waiting area with a check-in desk for adults to register and communicate needs with clinic staff, materials to help children and families get acclimated to the building and distract from the procedures while clinic staff assessed potential needs of the vaccine recipient; (2) private cubicles for quiet vaccine administration, with as-needed clinical support; and (3) a post-vaccine waiting area with snacks, water, and games/toys.

The clinical, behavioral, and environmental supports provided to autistic individuals and persons with other IDD to facilitate their obtaining the COVID-19 vaccine were based on cognitive-behavioral practices. Examples of clinical interventions throughout the vaccine experience included guided transition time, access to visual supports including a map of the museum’s attractions, a social story detailing steps of the SFVC experience, distractions like fidget toys and children’s coloring pages, stress-reducing activities, relaxation strategies, cognitive restructuring/“helpful thoughts” and scaffolded exposure. Specific strategies were implemented according to clinical judgement and need, and therefore varied per person. Per the Centers for Disease Control and Prevention (CDC) general best practice guidelines for immunization, participants were required to sit for 15 min after receiving vaccination (CDC, 2022a, 2022b). During this time, water, snacks, and toys were available, and the individuals and their caregivers were invited to participate in a survey about their current vaccine clinic experience. After the post-vaccination period, participants and their family members received free entry to the ANS museum prior to opening to the public, for a sensory-friendly experience.

Outreach efforts to invite people to attend the SFVC events included social media posts to AJDAI accounts, email blasts to AJDAI listservs (comprising more than 1500 past research participants, employees, and community stakeholders, and individuals who expressed interest in learning more about the AJDAI), targeted communication to community groups that serve autistic populations, and a press release through a local news outlet coverage for the first SFVC. Vaccination appointments were scheduled in advance, although walk-in vaccinations were welcome. The SFVC provided vaccines to all family members and caregivers, including children and adults. Additionally, staff engaged in retention efforts by assisting vaccine recipients and their families in scheduling appointments for their future COVID-19 vaccine doses while onsite at the SFVC.

### Participants

All participants who intended to receive a vaccination at a SFVC, totaling 157 individuals, received one or more vaccines during 7 SFVC sessions. Although the SFVC was marketed towards autistic individuals and persons with other IDD, vaccines were also provided to caregivers and individuals without autism/IDD who accompanied autistic individuals and persons with other IDD on the day of the SFVCs. However, study participation was limited to one informant (self or caregiver) reporting on vaccination for autistic individuals and persons with IDD (i.e., a caregiver who also got a vaccine did not complete a survey about their own vaccine experience, only that of their eligible child). Among total individuals vaccinated, data was not recorded on how many were eligible for the study. The survey sample consisted of 35 adults (22.3% of the total population who received at least one vaccine at a SFVC), all of whom were either an autistic individual or person with other IDD (n = 5) or legal guardian of an autistic individual or person with other IDD (n = 30). Survey questions asked about the vaccine recipient’s experience. Caregiver perspective on their child’s experience was attained when completing it on their behalf. Diagnoses were self- or caregiver-reported. Participants included any person at the SFVC who consented to and completed any portion of the survey. Survey sample size is partially reflective of barriers to survey completion at the clinic (e.g., opting out, choosing to complete via QR code later and never accessing it, or technology restrictions that imposed too great of a time commitment). Of the 35 respondents, 32 agreed to be contacted in the future for additional questions and 18 participated in a brief supplemental interview.

Inclusion criteria consisted of an individual (a) with intellectual or developmental disability (IDD) including autism and/or their caregiver, who was eligible to receive a COVID-19 vaccine, (b) who attended a SFVC and attempted to receive any dose of the COVID-19 vaccine, (c) aged 4–90 years, and (d) with sufficient English fluency to complete the questionnaire. Adults unable to consent and minors had a legal guardian consent to participation and provided verbal assent. Exclusion criteria included an individual who (a) attended the SFVC, open to the public, but was not an autistic individual or caregiver of an individual with autism or other IDD or (b) was not fluent in English.

#### Survey and Interview Methods

Attendees at the SFVCs were invited to complete an electronic survey. The survey was completed during the waiting period after vaccine administration on an iPad, or later via QR-code access. Access to the survey terminated after the final SFVC.

At the end of the survey, participants were asked if they would be willing to be contacted by study staff for an additional interview to gather more information about their experiences. Those who agreed were contacted by study staff up to two times by either phone call/voicemail, or text message. Respondents were then scheduled for a Zoom or phone call for a 15-min supplemental interview. Two research staff members conducted interviews. Staff members followed an interview script (see Appendix B) that expanded on the open-ended questions asked in the initial Qualtrics survey.

### Measures

#### Vaccine Experience/History Survey Questions—Likert Scale (See Appendix A)

Likert scale questions included a 5-point scale: strongly disagree, disagree, neutral, agree, and strongly agree. The 8 Likert scale questions addressed the respondent’s opinions on the (1) physical environment of the SFVC, (2) clinical expertise at the SFVC, (3) clinicians’ response to SFVC attendees’ questions and concerns, (4) helpfulness of the SFVC compared to previous attempts elsewhere, (5) visual supports at the SFVC, (6) opportunity to explore the ANS museum post-vaccine, (7) recommending the SFVC to others with similar needs, and (8) retention to future SFVC.

#### Vaccine Experience/History Survey Questions—Open-Ended Questions (See Appendix B)

The survey included 8 open-ended questions, which were repeated and/or elaborated on during supplemental interviews and included (1) how participants learned of the SFVC, (2) most helpful parts of the clinic, (3) least helpful parts of the clinic, (4) suggestions for improvement for future SFVCs, (5) descriptive factors of the autistic individual or individual with IDD receiving the vaccine, (6) information regarding previous attempts to receive the COVID-19 vaccine, (7) information regarding successful COVID-19 vaccine attempts elsewhere, and (8) any additional information. Responses from these questions were coded into common themes.

#### Demographics

Demographic questions included race, ethnicity, age, sex, diagnoses, relationship to survey respondent, vaccine dose received at time of survey administration, and verbal ability of the autistic individual or individual with other IDD.

#### Supplemental Interview

Interview scripts were tailored based on participant responses previously provided via Qualtrics (e.g., if participant provided the response “staff was very knowledgeable about autism” in response to a “what was most helpful about the SFVC?” question via Qualtrics, the staff interviewer would address the response in the interview by stating, “You have previously noted that the most helpful part of the clinic was that the staff was very knowledgeable about autism. Is there anything else you would like to add or expand on for most helpful parts of the clinic?”) Interviews were recorded and then transcribed, to ensure an accurate recording of responses.

### Procedures and Data Analytic Plan

All survey data was gathered using Qualtrics. To characterize results of sociodemographic questions, descriptive statistics were computed. Survey questions using the Likert scale were analyzed as quantitative data using Statistical Package for the Social Sciences (SPSS), version 28.0. Open-ended text responses on survey questions and interview responses were thematically organized and examined qualitatively using NVivo 12 software. Qualitative content was coded through an inductive approach, which is part of the grounded theory of qualitative coding. A codebook was created by coders to represent sub-themes. The codebook along with illustrative quotes/participant responses is displayed in Table 4.

#### Intercoder Reliability

Intercoder reliability for qualitative data between the two main coders was calculated using single proportional agreement. Both coders completed all responses. Reliability was calculated by taking the number of code agreements (238), divided by the total number of code agreements and disagreements (255) and multiplying by 100. Agreement was 93%. Single proportional agreement was used based on several hindering factors that would not allow for more complex statistics, including large number of codes and the possibility for multiple codes on a text unit (Campbell et al., 2013). A third independent coder reviewed the disagreements between coder 1 and coder 2 and the majority code was recorded.

## Results

### Demographics

Thirty-five survey respondents reported demographic information on themselves, their family, and the vaccine recipient. Respondents reported learning of the SFVC via email (34.3%), (social) media (34.3%), or word of mouth (28.6%). Respondent demographics are displayed in Table 1. Demographics categorizing the autistic individual or person with other IDD receiving the vaccine are displayed in Table 2. Respondents were most often the legal guardian of the vaccine recipient (85.7%). Most vaccine recipients were male (65.7%). They ranged in age from 4 to 28 years old, with the 4–10 years age-range represented most frequently (45.75%). Race was most frequently reported as White (34.3%), and most recipients were non-Hispanic/Latine (65.7%).

**Table 1:** Respondent demographics

Respondent demographics (n = 35)	n (%)
Gender	
Male	5 (14.3%)
Female	19 (54.3%)
Transgender male	–
Transgender female	–
Non-binary	–
Gender-diverse	–
Not reported	11 (31.4%)
Relationship to participant	
Legal guardian	30 (85.7%)
Self	5 (14.3%)
Family income (per year)	
$0–$50,000	9 (36.0%)
$50,000–$99,000	1 (2.9%)
$100,000+	12 (34.3%)
Not reported	13 (37.1%)

**Table 2:** Characterizing participants who were vaccine-recipients

Characterization (n = 35)	n (%)
Developmental diagnoses	
Autistic only	12 (35.3%)
Other IDD only	1 (2.9%)
2 diagnoses	4 (11.4%)
3 + diagnoses	8 (22.9%)
Not reported	11 (31.4%)
Vaccine dose (at time of survey)	
Dose 1	5 (14.3%)
Parti Dose 2	9 (25.7%)
Booster (Dose 3 or 4)	3 (8.6%)
Not reported	18 (51.4%)
Gender	
Male	23 (65.7%)
Female	5 (14.3%)
Transgender male	–
Transgender female	–
Non-binary	–
Gender-diverse	–
Not reported	7 (20.0%)
Age in years	
4–10	16 (45.6%)
11–20	8 (22.9%)
21–30	4 (11.4%)
Not reported	7 (20.0%)
Race	
White	12 (34.3%)
Black or African American	7 (20.0%)
Asian	3 (8.6%)
Native Hawaiian or other Pacific Islander	2 (5.7%)
More than 1 race	3 (8.6%)
Not reported	8 (22.9%)
Ethnicity	
Non-Hispanic/Latine	23 (65.7%)
Hispanic/Latine	5 (14.3%)
Not reported	7 (20.0%)
Verbal ability	
Verbally fluent	19 (54.3%)
Minimally verbal	6 (17.2%)
Non-verbal	2 (5.7%)
Not reported	8 (22.8%)

IDD refers to participants with intellectual or developmental disability. Verbally fluent was defined as verbal speech; minimally verbal was defined as some single words or word approximations; and non-verbal was defined as sounds or utterances only

### Likert-Scale Responses

Of the 32 respondents who completed the Likert-scale section of the survey, 23 completed all eight questions, seven opted out of answering one item, and two opted out of answering two items. Distributions of responses are displayed in Table 3. Participants most often reported strongly agree for all questions, suggesting that the overall experience of the SFVC was positive, promoted retention, and would be recommended to other autistic individuals or persons with other IDD.

**Table 3:** Ratings of SFVC experiences based on Likert scale results

Scale ratings	1		2		3		4		5		NR	
Item content	n	%	n	%	n	%	n	%	n	%	n	%
1. Physical space met family needs	2	6.3%	–	–	1	3.1%	8	25%	21	65.6%	–	–
2. Knowledgeable clinicians	1	3.1%	–	–	–	–	5	15.6%	26	81.3%	–	–
3. Questions answered clearly	2	6.3%	–	–	1	3.1%	5	15.6%	22	68.8%	2	6.3%
4. More helpful than other clinics	2	6.3%	–	–	1	3.1%	2	6.3%	24	75.0%	3	9.4%
5. Helpful visual supports	1	3.1%	1	3.1%	3	9.4%	4	12.5%	21	65.6%	2	6.3%
6. Influence of museum access	3	9.4%	1	3.1%	4	12.5%	5	15.6%	18	56.3%	1	3.1%
7. Would Recommend SFVC	1	3.1%	–	–	1	3.1%	2	6.3%	27	84.4%	1	3.1%
8. Intention to return to SFVC	1	3.1%	1	3.1%	2	6.3%	1	3.1%	25	78.1%	2	6.3%

Scale Ratings refer to the different response options provided on the survey: Strongly Disagree (1), Disagree (2), Neutral (3), Agree (4), Strongly Agree (5). No Response (NR) indicates no response recorded. Refer to Appendix A for a list of full items

### Open-Ended Responses

Based on Qualtrics participant-report and supplemental interview data, several themes were identified; see Table 4. Responses were further categorized into reported facilitators, barriers, and suggested improvements to successful vaccine administration.

**Table 4:** SFVC open-ended survey and supplemental interview responses

Code	Description	Illustrative response	Responses (n =)
*Most helpful parts of the clinic (Facilitators)*	Elements of the clinic that were most helpful		
Environment	Refers to the clinic’s general environmental influence	“Enjoyed the atmosphere”	n = 17
Environmental stimuli	Refers to components of the clinic’s environment that positively impacted participants or had a sensory influence	“Quiet environment, calm”	n = 4
Location	Positive references to the clinic’s location (both being at the ANS museum and geographical location in center city, Philadelphia)	“Even without a car easy to access and get to”	n = 14
Sensory-friendly elements	Components of the clinic that supported sensory needs	“Sensory component and fewer people there made him feel less nervous because it's typically visually stressful in the area, and with ANS there weren't a lot of people and less sound which are sensitive areas”	n = 22
During vaccination	Sensory accommodations that were used during the actual COVID-19 vaccine administration	“Separate room”	n = 4
Waiting periods	Sensory accommodations that were used during waiting periods (e.g., sign-in, post-vaccine)	“Snacks and fidget toys during waiting period was really helpful”	n = 6
Staff clinical expertise	Refers to the clinic’s staff and their efforts to support participants in an adaptable and sensitive way	“It was so nice to be surrounded by people who understood parent and child. The plan that the clinician provided for my son was extremely helpful, explaining each level of fear and stress while getting to the actual injection point”	n = 22
Timeline	General flow and timeline components referencing event(s) occurring during the clinic (e.g., quick check-in process)	“Early start time, easy access”	n = 12
Scheduling	Any reference to scheduling/registration or clinic availability (e.g., occurring on weekends, easy online registration, open appointments)	“Able to go online and book reservation for a time that worked”	n = 6
Vaccine experience	Successful components about the clinic that impacted the participant’s entire vaccine experience	“Smooth, quick registry to vaccine process, no waiting meant less time for child's anxiety to build”	n = 7
*Least helpful parts of the clinic (Barriers)*	Elements of the clinic that were least helpful		
Environmental stimuli	Refers to components of the environment at the clinic (e.g., clinic space, visuals)	“There was a visual of a character receiving a needle as you entered the clinic. The child saw this visual and got nervous”	n = 2
Logistics	Refers to logistical components of the clinic’s operations	“Hard to find location”	n = 8
Family support	Logistical elements related to family ease (e.g., parking, childcare)	“[Mother] had to bring all 3 children. Childcare would have been helpful”	n = 2
Vaccine experience	General logistic components about the participant’s COVID-19 vaccine experience (e.g., scheduling difficulties, not enough staff support, impact on children)	“Too much time was given for the child to get comfortable. This may have led to more anxiety for the child leading up to the shot”	n = 6
*Improvements to make*	Participant ideas for improving future SF vaccine clinics		
Activity variation	Increasing variety of activities present for participants to engage in	“Extend the variety of activities. Have live entertainment, more visual distractions, different strategies for different learners. Fine motor activities for my son was difficult, so coloring was not a good option for him”	n = 3
Communication	Vaccine clinic can be improved through additional communication to families and participants	“Parking directions would be helpful—where to park and free parking”	n = 7
Advertising to the community	Communication with a focus on expanding advertisement and awareness in the community	“If trying to cater to ASD in Philly, could advertise more (ex: Facebook) and tailor to demographic and try and get as many people there as possible”	n = 3
Family eligibility	Communication to families with a focus on sharing information regarding vaccine eligibility for people other than people with special needs	“Parents did not know they could get the vaccine as well—advertise this possibly!”	n = 1
Logistics and environment	Improvements made to the logistics (e.g., flow, organization) and environment (e.g., sensory stimuli, location) of the clinic	“Maybe have a person in front at desk/entrance area to guide instead of museum employee”	n = 12
Non-COVID-19 vaccines	Improvements centered around providing non-COVID-19 vaccines at the clinic	“Perhaps give other vaccines too, like the flu shot”	n = 1
*Other notes*	Any additional comments made that did were not part of the main interview categories		
Museum	Comments made in reference to the ANS museum (e.g., open space, access to visit)	“Having tickets for the museum afterwards gave the child something to look forward to”	n = 11
Retention	Comments made about the desire for upcoming clinics or multiple uses of the clinic	“Came for both vaccine doses”	n = 5
*Unsuccessful vaccine attempts elsewhere*	COVID-19 vaccine attempt at another vaccination		
Vaccine NOT administered	COVID-19/other vaccine was NOT successfully administered at a location other than the Drexel SF Vaccine clinic	“Many unsuccessful attempts at Dr.’s office”	n = 5
Behavioral challenges	Reference to child/participant behavioral challenges preventing successful vaccine administration	“Son ended up causing a scene, and we left”	n = 1
Environmental factors	Reference to vaccine barriers caused by ill equipped pharmacy/clinic	“The room was very crowded”	n = 3
*Vaccine administered elsewhere*	COVID-19/other vaccine was successfully administered at a location other than the Drexel SF Vaccine clinic		
Negative experience	Vaccine was successfully administered another location, but the experience was negative (e.g., participant had complaints about staff, vaccine experience, etc.)	“Received vaccine elsewhere but had a negative experience.”	n = 2
Restraint	Vaccine was successfully administered at another location but required the use of restraint (e.g., physically holding the participant down)	“Had to hold old my son down to receive the vaccine”	n = 2
Sensitive pharmacy staff	Vaccine was successfully administered at another location, staff was reported to be sensitive (e.g., supported the participant, displayed clinical expertise)	“Clinicians were narrating what was happening. Clinicians who exude calmness also keep children calm”	n = 3
Sensory friendly clinic (not AJDAI)	Vaccine was successfully administered at another location that was noted to be “sensory friendly” (e.g., provided support for neurodiverse individuals/individuals with sensory sensitivities)	“Lincoln financial field with the Eagles so it was enticing. It was also a sensory meet up but was more crowded and not as conveniently located as ANS”	n = 1

#### Facilitators at SFVC

All participants reported facilitators to successful vaccine administration at the SFVC. Respondents most frequently identified staff clinical expertise (n = 22) as a primary facilitator, referring to the clinic’s staff and their efforts to support participants in an adaptable and sensitive way. Respondents also identified sensory friendly elements (n = 22), as a facilitator to successful vaccination. Sensory friendly elements refer to components of the clinic that supported sensory needs, such as low-noise stimuli or less crowded environments, illustrated by the respondent quote, “Sensory component and fewer people there made him feel less nervous because it's typically visually stressful in the area, and with ANS there weren’t a lot of people and less sound which are sensitive areas.” Respondents also reported that the clinic’s location (both being at the ANS museum and geographical location in Center City, Philadelphia) facilitated access to vaccination. Refer to Table 4 codes under the section “most helpful parts of the clinic.”

Additionally, several responses mentioned access to the museum as a specific facilitator (n = 11), as illustrated by a participant quote “having tickets for the museum afterwards gave the child something to look forward to.” Participant retention was also anecdotally reported. Some participants indicated that they “came for both vaccine doses,” suggesting positive experiences with the current SFVC and the staff that administered the vaccines. Refer to Table 4 codes under the section “other notes.”

#### Facilitators at Clinics Other than SFVC

Seven respondents reported that they had received prior doses of COVID-19 vaccination at locations other than the current clinic. Four of these respondents identified facilitators to successful vaccine administration, stating that sensitive pharmacy staff and attending another clinic that was identified as sensory-friendly aided in the positive experience. Refer to Table 4 codes under the section “vaccines administered elsewhere.”

#### Barriers at SFVC

None of the autistic individuals or persons with other IDD who came to the SFVC left without a successful vaccination, suggesting that the SFVC provided adequate adaptations and accommodations to mitigate common barriers to successful vaccine administration. Most participants (n = 26) reported that there were no unhelpful parts of the current clinic. Of participants that identified unhelpful aspects of the current clinic, the most common theme (n = 6) referred to the general logistic components of the participant’s COVID-19 vaccine experience (e.g., scheduling difficulties, parking, not enough staff support, logistics impact on children). These results suggest that although the vaccine was successfully administered, the general logistic components about the participant’s vaccine experience could serve as a potential barrier to future successful vaccine experiences. The logistical components may have been an inconvenience to care but did not necessarily serve as a barrier to successful vaccine administration. Refer to Table 4 codes under the section “least helpful parts of the clinic.”

#### Barriers at Clinics Other than SFVC

Of the 7 respondents that identified successfully receiving a previous dosage of the COVID-19 vaccine elsewhere, 4 responses were classified as negative experiences. Two respondents mentioned that restraint was used at the other clinic. Five respondents disclosed experiencing prior unsuccessful COVID-19 vaccine attempts at locations other than the SFVC. Survey respondents identified environmental factors as barriers, which were further described as barriers caused by ill-equipped pharmacy/clinics that were not sensitive to the sensory needs of the participant with special needs. A respondent stated that “the room was very crowded.” Respondents also noted that behavioral challenges inhibited successful vaccine administration, illustrated by the quote “[my] son ended up causing a scene, and we left.” Refer to Table 4 codes under sections “vaccines administered elsewhere” and “unsuccessful vaccine attempts elsewhere.”

#### Suggested Improvements for Successful Vaccine Administration

Participants identified several areas of improvement for future SFVCs. The most frequently identified area of improvements included improving the overall logistic components or “flow” of the clinic, making it easier for families to navigate (n = 12), illustrated by the respondent quote “Maybe have a person in front at desk/entrance area to guide instead of museum employee,” referring to the need for more guidance by clinical staff opposed to museum staff. Additional identified areas of improvement were aimed at increasing the variety of distracting activities in waiting areas for autistic individuals or individuals with other IDD, expanding on communication to caregivers and parents, and amplifying marketing to reach more families with autistic individuals, as well as expanding the reach of these clinics to non-COVID-19 vaccines, such as the annual flu vaccine. These results suggest that having seamless operations, increased diversity of distracting activities during vaccine administration and waiting periods, consistent communication, and increased advertising to community members could serve as potential facilitators for future vaccine clinics. Refer to Table 4 codes under the section “improvements to make.”

## Discussion

Findings from this survey of caregivers and autistic individuals or persons with other IDD who received one or more COVID-19 vaccines at our SFVCs suggest that there are several practices that facilitate vaccination. Respondents highlighted the impact of clinically experienced staff with expertise in autism, activities available during the waiting period, and neutral or even positive non-medical association with the site venue.

Findings from these SFVCs are consistent with previously identified barriers and facilitators to preventative healthcare (O’Hagan et al., 2023; Ryerson et al., 2021) and best practices to support autistic individuals and persons with other IDD in receiving vaccines (Call et al., 2022; Pavlov et al., 2023), while also highlighting novel facilitators that may assist this population in accessing and receiving preventative healthcare. Results that align with previously identified facilitators include the endorsement of staff clinical expertise/clinicians equipped with autism specific training, as well as flexibility of care (O’Hagan et al., 2023). Previous research found completing pre-visit questionnaires on the vaccine recipient’s past medical experiences, sensory sensitivities, behavior, and preferred items to be successful (Pavlov et al., 2023); however, this study did not include an informational interview prior to the vaccine visit. Results that replicate previously identified barriers to successful vaccine experiences include logistic factors such as transportation difficulties, parking availability, communication with families, and securing appointments (Ryerson et al., 2021). Novel facilitators identified include hosting the SFVC at a neutral location (free from any medical exam history), having a something to look forward to/a “reward” after the completion of the vaccine (access to the museum), consistency of location and staff administering the vaccine, as well as sensory-friendly elements during and after the vaccine was administered. Importantly, survey responses suggest satisfaction with the SFVC, evidenced by most participants indicating an intention to recommend the clinic to others and return to the clinic themselves. Additional responses indicate that participants thought clinicians were knowledgeable and that the clinic was more helpful than other clinics attended in the past. Survey responses provide immense support for the need and benefit of sensory friendly vaccine clinics to help the autistic population access quality healthcare. Findings suggest that having trained, high-quality staff/clinicians, sensory-friendly elements, and hosting the location of the vaccine clinic in a neutral (e.g., no medical exam history) or accessible location associated with recreation or entertainment are facilitators for successful vaccine administration.

Few participants identified barriers to care at the SFVC, suggesting that accommodations and adaptations implemented were helpful in supporting successful vaccine experiences. Barriers reported included logistic factors (e.g., scheduling difficulties, family support and communication, etc.) and are consistent with previous research (Ryerson et al., 2021), although these barriers did not impede successful vaccination. This emphasizes the need for future researchers and clinicians to address logistical barriers in a proactive manner.

Recommendations for future SFVCs include having behaviorally trained, high-quality staff/clinicians present at the clinic to engage in preventative strategies to reduce challenging behavior in response to vaccine attempts. It is recommended that trained staff engage in behavior-based strategies such as modeling, high probability request sequences, providing choices, and noncontingent reinforcement (Call et al., 2022). It is also recommended that future SFVCs incorporate sensory-friendly elements like reducing aversive sensory stimuli (Call et al., 2022). Additional recommendations based on survey findings include hosting the location of the vaccine clinic in a neutral (e.g., non-medical) or accessible location associated with recreation or entertainment are facilitators for successful vaccine administration, establishing multiple streams of communication with vaccine recipients and their families, keeping communication clear and consistent, and creating a flexible schedule of SFVCs that meet the needs of individuals served (e.g., offer weekday clinics, evening clinics, etc.). If hosting a clinic at a neutral site is not feasible, efforts can be directed to providing materials and processes (e.g., visual tour of clinic space, step-by-step outline of the vaccination procedures, pictures of clinic staff) and recommendations in advance to help families prepare children for vaccination. When resources required for specialized clinics are not attainable, there is opportunity to embed designated sensory-friendly clinic hours, equipped with behaviorally trained clinical staff (e.g., psychologists, behavior analysts) into hospital/clinic standard operations. Specialized vaccination clinics within hospital settings were noted to be highly acceptably among caregivers (Pavlov et al., 2023), which provides a sustainable approach given that these settings are where most healthcare is received. Importantly, training programs for medical staff (e.g., medical doctors, nurses, pharmacists, medical assistants) who administer vaccinations daily as part of their duties may also assist in facilitating more successful vaccine experiences. Additionally, ongoing consultation could provide medical staff with skills to implement iterations of sensory-friendly procedures directly and independently, based on feedback from attendees and other metrics of success in reducing barriers for vaccination of autistic individuals. This model of training would involve a large initial response effort, which fades once medical staff are trained to fidelity. Training packages could consist of behavior skills training (BST), an evidence-based practice consisting of four parts (a) instruction, (b) modeling, (c) rehearsal, and (d) feedback. BST could be used to teach explicit skills to medical staff working with autistic individuals or persons with other IDD.

Future SFVC methods of recruitment should be expanded upon to assist in increasing public awareness and subsequent vaccination accessibility, through methods like social media advertisement, postings at community centers/forums, and providing clinic information to healthcare personnel (e.g., pediatricians, occupational therapists, behavioral therapists, speech pathologists) likely to be seen by a variety of potential participants. Such practices could reduce health disparities related to disability beyond the COVID-19 pandemic. More information about autistic individuals’ and persons with other IDD’s vaccine experiences is needed and could inform best practices for those providing care for this population.

### Limitations

There were several limitations to this study. Due to the small sample size, findings may not be generalizable to all autistic individuals or their caregivers, and associations between age and responses were not evaluated. Selection bias was a key limitation in this study. Participants were recruited to attend the SFVCs through various outreach methods, however, recruitment relied heavily upon existing AJDAI relationships, such as membership on a listserv, following on social media, or an AJDAI community partner. Therefore, many SFVC attendees may have been familiar or interacted with AJDAI in the past and potential eligible attendees not previously familiar with AJDAI may have been missed. Furthermore, among those who attended the SFVC, participants volunteered to participate in surveys and supplemental interviews. Participants with certain characteristics (e.g., communication difficulties, limited time, mistrust in research, etc.) may have been less likely to volunteer compared to others. Participants also needed to travel to the vaccine clinic site. Therefore, individuals with greater support needs, such as individuals from residential facilities, did not participate. In addition, data were primarily from caregivers, with few responses directly from autistic individuals or those with IDD. Future research should focus on more inclusive efforts to encourage responses from non-English speakers and those with low socioeconomic status. Additional efforts should focus on including more autistic voices to self-report their feedback instead of solely relying on caregiver or legal guardian reports to help ensure autistic individuals are being fairly represented and sufficiently included. Another limitation includes the lack of input from healthcare providers that routinely serve this population, as well as healthcare administrators that may have influence over incorporating similar clinics in their settings. Lastly, this study collected limited data about relevant clinic details which creates opportunities for future research to further explore related topics. Measures should be adapted to incorporate information about the duration of clinic visits, number of personnel required to support successful vaccinations, number of people who reoccur at the clinic for multiple doses/vaccines, and the types of supports used most frequently by clinic staff.

## Conclusion

All individuals who came to a SFVC to receive a COVID-19 vaccine were administered a vaccine successfully. While barriers to care were consistent with previous literature, novel facilitators were identified. Future research should amplify autistic voices, incorporate feedback to identify potential barriers to access to care, as well as expand on facilitators to create a more positive vaccine experience for autistic persons or individuals with other IDD. Continued data from successful and unsuccessful vaccine experiences may inform a future standard of care for providing preventative healthcare for autistic individuals or persons with other IDD.

## Appendix A

### Likert-Scale Survey Questions about SFVC Experience

1. THINK ABOUT YOUR MOST RECENT VACCINE CLINIC EXPERIENCE. Please assess your rating for the following statements. Think about anyone in your family who received a vaccine at the most recent clinic.—The physical space and layout of the clinic area met my/my family's needs
2. THINK ABOUT YOUR MOST RECENT VACCINE CLINIC EXPERIENCE. Please assess your rating for the following statements. Think about anyone in your family who received a vaccine at the most recent clinic.—Clinicians were knowledgeable and responsive to my/my family's needs
3. THINK ABOUT YOUR MOST RECENT VACCINE CLINIC EXPERIENCE. Please assess your rating for the following statements. Think about anyone in your family who received a vaccine at the most recent clinic.—My/my family's questions were answered clearly by the vaccine team
4. THINK ABOUT YOUR MOST RECENT VACCINE CLINIC EXPERIENCE. Please assess your rating for the following statements. Think about anyone in your family who received a vaccine at the most recent clinic.—This sensory friendly clinic experience was more helpful than previous vaccine clinic experiences
5. THINK ABOUT YOUR MOST RECENT VACCINE CLINIC EXPERIENCE. Please assess your rating for the following statements. Think about anyone in your family who received a vaccine at the most recent clinic.—The visual supports (including handouts and social story) were helpful
6. THINK ABOUT YOUR MOST RECENT VACCINE CLINIC EXPERIENCE. Please assess your rating for the following statements. Think about anyone in your family who received a vaccine at the most recent clinic.—The opportunity to tour the Museum after the shot influenced our decision to attend this clinic
7. THINK ABOUT YOUR MOST RECENT VACCINE CLINIC EXPERIENCE. Please assess your rating for the following statements. Think about anyone in your family who received a vaccine at the most recent clinic.—I would recommend this clinic to other individuals with special needs and their families
8. THINK ABOUT YOUR MOST RECENT VACCINE CLINIC EXPERIENCE. Please assess your rating for the following statements. Think about anyone in your family who received a vaccine at the most recent clinic.—I intend to return to this clinic again

## Appendix B

### Open-Ended Survey and Interview Questions

1. *Confirm whether most recent clinic was sensory-friendly or other clinic. If sensory-friendly:* Where/how to you hear about this clinic?
2. What components of the clinic did you find most helpful? *Alternative if participant completed text box response on survey: You noted that you found [insert text based on survey responses] helpful. Do you have any more information you want to share?*
3. What components of the clinic did you find least helpful? *Alternative if participant completed text box response on survey: You noted that you did not find [insert text based on survey responses] helpful. Do you have any more information you want to share?*
4. What suggestions do you have that would improve your vaccine clinic experience in the future? *Alternative if participant completed text box response on survey: You noted [insert text based on survey responses] suggestions. Do you have any more information you want to share?*
5. *Adapt based on information provided in survey:* Describe the autistic person or person with IDD whose experience receiving the COVID vaccine you are sharing today (age, sex, verbal ability, level of supports required, attending school or educational program outside the home, state of residence)
6. If participant endorsed unsuccessful attempts to be vaccinated in the past: Please tell me more about the other times you tried to receive the COVID vaccine for [person with special needs]. How did that impact you and your family? What supports would have been helpful? What factors made this attempt unsuccessful?
7. *Adapt based on information provided in survey – may not be needed if covered in items 2–4:* Describe the visit where you were able to get the COVID vaccine. What was different there from the previous attempts, if any? What helped succeed in getting the vaccine? Were any supports and/or accommodations planned ahead of time or were they figured out in the moment? Was there a clinician or staff member that was specifically trained to work with individuals with autism or other special needs?
8. Is there anything else you would like us to know?

*Note.* Sections of questions written in italics indicate adaptations to questions that the interviewer could have made depending on what information was already gathered from the survey.
